# Intramyocardial transplantation of cardiac mesenchymal stem cells reduces myocarditis in a model of chronic Chagas disease cardiomyopathy

**DOI:** 10.1186/scrt470

**Published:** 2014-07-01

**Authors:** Daniela Nascimento Silva, Bruno Solano de Freitas Souza, Carine Machado Azevedo, Juliana Fraga Vasconcelos, Rejane Hughes Carvalho, Milena Botelho Pereira Soares, Ricardo Ribeiro dos Santos

**Affiliations:** 1 Centro de Biotecnologia e Terapia Celular, Hospital São Rafael, Av. São Rafael, 2152. São Marcos 41253-190, Salvador, BA, Brazil; 2 Laboratório de Engenharia Tecidual e Imunofarmacologia, Centro de Pesquisas Gonçalo Moniz, Fundação Oswaldo Cruz, Avenida Waldemar Falcão, 121, Candeal, Salvador 40296-710, BA, Brazil

## Abstract

**Introduction:**

New therapeutic options are necessary for patients with chronic Chagas disease, a leading cause of heart failure in Latin American countries. Stem cell therapy focused on improving cardiac function is a promising approach for treating heart disease. Here, we evaluated the therapeutic effects of cardiac mesenchymal stem cells (CMSCs) in a mouse model of chronic Chagas disease.

**Methods:**

CMSCs were isolated from green fluorescent protein (GFP) transgenic C57BL/6 mouse hearts and tested for adipogenic, osteogenic, chondrogenic, endothelial, and cardiogenic differentiation potentials evaluated by histochemical and immunofluorescence techniques. A lymphoproliferation assay was performed to evaluate the immunomodulatory activity of CMSCs. To investigate the therapeutic potential of CMSCs, C57BL/6 mice chronically infected with *Trypanosoma cruzi* were treated with 10^6^ CMSCs or saline (control) by echocardiography-guided injection into the left ventricle wall. All animals were submitted to cardiac histopathological and immunofluorescence analysis in heart sections from chagasic mice. Analysis by quantitative real-time reverse transcription polymerase chain reaction (qRT-PCR) was performed in the heart to evaluate the expression of cytokines involved in the inflammatory response.

**Results:**

CMSCs demonstrated adipogenic, osteogenic, and chondrogenic differentiation potentials. Moreover, these cells expressed endothelial cell and cardiomyocyte features upon defined stimulation culture conditions and displayed immunosuppressive activity *in vitro*. After intramyocardial injection, GFP^+^ CMSCs were observed in heart sections of chagasic mice one week later; however, no observed GFP^+^ cells co-expressed troponin T or connexin-43. Histopathological analysis revealed that CMSC-treated mice had a significantly decreased number of inflammatory cells, but no reduction in fibrotic area, two months after treatment. Analysis by qRT-PCR demonstrated that cell therapy significantly decreased tumor necrosis factor-alpha expression and increased transforming growth factor-beta in heart samples.

**Conclusions:**

We conclude that the CMSCs exert a protective effect in chronic chagasic cardiomyopathy primarily through immunomodulation.

## Introduction

Heart disease remains a major cause of worldwide morbidity and mortality. Despite advances in clinical and surgical care of cardiac patients, current therapies are able to treat symptoms, delay clinical deterioration, and increase survival but are not effective in repair induction in a diseased heart. This is the case of chronic cardiac Chagas disease, which is caused by the protozoan parasite *Trypanosoma cruzi* and remains a leading cause of heart failure in Latin America [1]. Therefore, a major effort is under way to develop therapies aiming at regenerating the myocardium or to stimulate endogenous repair programs.

Different cell types, such as bone marrow cells, mesenchymal stem cells (MSCs) from adipose tissue, and skeletal myoblasts, have been tested in basic and applied clinical studies [1-4]. Bone marrow cells have demonstrated limited efficacy in many clinical trials, and this has raised the question of its usefulness as well as increased the investigation of other stem cell sources that may be potentially more effective in heart disease treatment. Moreover, different cell types are likely to have therapeutic potential in various disease settings, depending on the particular cardio-pathogenic mechanisms involved.

Adult cardiac stem cell populations have previously been observed in both murine and human hearts, including tissue-specific MSCs [5-14]. Owing to their multipotentiality and direct action via secretion of a repertoire of molecules that stimulate tissue regeneration and immunomodulation, MSCs have been used in different clinical trials and experimental models that reproduce tissue damage in order to verify their therapeutic potential [15-19].

In this study, we isolated a population of stem cells from the heart tissue that displays cell phenotype and immunosuppressive and differentiation potentials characteristic of MSCs. The therapeutic potential of these cells was evaluated in an experimental model of chronic Chagas disease cardiomyopathy.

## Materials and methods

### Animals

Male C57BL/6-Tg(CAG-EGFP)1Osb/J (The Jackson Laboratory, Bar Harbor, ME, USA) mice (4 to 8 weeks old) were used to obtain cardiac mesenchymal stem cells (CMSCs). Wild-type female C57BL/6 (4 weeks old) were used for *T. cruzi* infection and as non-infected controls. Animals were maintained in the animal facility of the Center for Biotechnology and Cell Therapy, Hospital São Rafael (Salvador, Bahia, Brazil), with access to food and water *ad libitum*. This study was approved by the local ethics committee for animal use at the Hospital São Rafael (CEUA-HSR).

### Isolation and culture of stem cells

Green fluorescent protein (GFP) transgenic mice were deeply anesthetized by using an inhaled isoflurane anesthetic circuit 2% (Abbott, Abbott Park, IL, USA) for 10 minutes and euthanized by cervical dislocation. The hearts were then removed to isolate CMSCs. Cardiac fragments (approximately 1 mm) were dissected and incubated with 0.1% collagenase type A (Sigma-Aldrich, St. Louis, MO, USA) at 37°C for 30 minutes under constant stirring. After chemical and mechanical dissociation, fragments were cultured in Dulbecco’s modified Eagle’s medium (DMEM) (Gibco, Grand Island, NY, USA) supplemented with 10% fetal bovine serum (FBS) (Gibco) and 1% Pen Strep (Gibco) as explants in sixwell plates and incubated at 37°C with 5% CO_2_. Culture medium was changed every 3 days, and on day 8, upon reaching 90% confluence, the explants were removed from the wells and adherent cells were trypsinized (Trypsin-EDTA 0.05%; Gibco) and transferred to tissue culture flasks containing medium supplemented with 10% FBS and 1% Pen Strep. The CMSC culture was maintained in a humidified incubator at 37°C with 5% CO_2_ for *in vitro* and *in vivo* studies.

### Phenotypic characterization by flow cytometry

CMSCs at passage 8 were trypsinized and resuspended in 0.9% saline. The cells (5 × 10^5^) were incubated for 5 minutes with CD16/CD32 (BD Biosciences, San Diego, CA, USA) with further incubation at 4°C for 30 minutes with the following antibodies (diluted at 1:100): Sca1-PE-Cy5.5 (Caltag Medsystems, Buckingham, UK); CD90.2-APC, CD117-PE, CD45-APC, CD34-Alexa Fluor 647, and CD44-PE (BD Biosciences); and CD29-APC and CD105-PE (BioLegend, San Diego, CA, USA). Isotype-identical antibodies were used as controls. After incubation and two phosphate-buffered saline (PBS) washes, the data were acquired and analyzed on an LSRFortessa flow cytometer (BD Biosciences). At least 50,000 events were collected and analyzed.

### Adipogenic, osteogenic, and chondrogenic differentiation

For adipogenic differentiation, cells were cultured in 24-well plates with 13-mm coverslips in complete medium (10^4^ cells per well). After reaching 50% to 60% confluence, the medium was removed and replaced with an adipogenic induction medium by using a StemPro Adipogenesis Differentiation Kit (Gibco). To observe the fatty vacuoles after 14 days in culture, the adipocyte differentiated cells and their controls were fixed in 4% paraformaldehyde and stained with Oil red solution. The images were captured by an AX70 microscope (Olympus, Tokyo, Japan) and ImagePro Plus 7.0 software (Media Cybernetics, San Diego, CA, USA). For osteogenic differentiation, the cells were cultured in a specific osteogenic differentiation medium by using a StemPro Osteogenesis Differentiation Kit (Gibco). Half of the differentiation medium was changed every two days. Calcium-rich matrix deposition was observed by staining with Alizarin red 2%. For chondrogenic differentiation, cells were cultured for 21 days in standard chondrogenic differentiation medium by using a StemPro Chondrogenesis Differentiation Kit (Gibco). Proteoglycan synthesis evaluation was performed in preparations stained with Alcian blue solution.

### Cardiomyogenic differentiation

CMSCs were cultured in DMEM and 10% FBS in 24-well plates with 13-mm coverslips with complete medium. After reaching 60% confluence, cells were incubated with 10 μM 5-azacytidine (Sigma-Aldrich). A control group was maintained in complete medium. After 24 hours of incubation, medium was replaced and cells were cultured in complete DMEM for an additional 4 weeks, when the expression of molecular cardiac markers was evaluated by immunofluorescence analysis. Cells were fixed with 4% paraformaldehyde for 30 minutes, washed twice with PBS for 3 minutes, and blocked with 5% bovine serum albumin in PBS for 30 minutes. The following primary antibodies were then applied and incubated at 4°C for 24 hours: anti-GATA-4 and anti-connexin 43 (1:200) (Santa Cruz Biotechnology, Inc., Santa Cruz, CA, USA). Images of differentiated cells and their controls were acquired by confocal microscopy (Olympus) and ImagePro Plus 7.0 software (Media Cybernetics).

### Endothelial cell differentiation

CMSCs were used for the endothelial cell induction protocol. Cells were seeded at a density of 3,000 cells per cm^2^ on 24-well plates in EGM-2 culture medium with supplements (Lonza, Walkersville, MD, USA) and cultured for 10 days. The expression of CD31, an endothelial cell marker, was then evaluated by immunofluorescence (BD Biosciences). On the following day, sections were incubated with Alexa fluor 594-conjugated anti-goat IgG (Molecular Probes, Carlsbad, CA, USA). To induce the formation of capillary-like structures, 24-well plates were covered with 250 μL of Matrigel (BD Biosciences) diluted 1:1 in EGM-2. CMSCs were seeded at a density of 30,000 cells per cm^2^ and cultured for 24 hours. The formation of capillary-like structures was observed over time by using an inverted microscope (Olympus). Additionally, the percentage of CD31 cells before and after EGM-2 induction was evaluated by flow cytometry with APC-conjugated anti-CD31 (BD Biosciences) diluted 1:50 and by analysis with the LSRFortessa flow cytometer.

### Lymphoproliferation assay

C57BL/6 spleen cell suspensions were prepared in RPMI medium (Invitrogen, Gibco-BRL, Gaithersburg, MD, USA) supplemented with 10% fetal bovine serum (Invitrogen, Gibco-BRL), 2 mM of L-glutamine, 0.1% RPMI 1640 vitamins solution (Sigma-Aldrich), 1 mM of sodium pyruvate, 10 mM of Hepes, 50 μM of 2-mercaptoethanol, and 50 μg/mL of gentamycin (Sigma-Aldrich). Splenocytes were cultured in 96-well plates at 4 × 10^5^ cells per well, in a final volume of 200 μL, in triplicate, in the presence of 5 μg/mL concanavalin A (Con A) only or in the presence of CMSCs pre-treated with 25 μg/mL mitomycin C, at different ratios (CMSC/splenocyte ratio of 1:1, 1:10, or 1:100). After 48 hours, plates were pulsed with 1 μCi of methyl-3 H thymidine (PerkinElmer, Amersham, Little Chalfont, UK) for 18 hours, and proliferation was assessed by measurement of 3 H-thymidine uptake by using a β-plate counter. The percentage inhibition of spleen cell proliferation was determined in relation to controls stimulated by Con A in the absence of CMSCs.

### *Trypanosoma cruzi* infection and cell therapy

Infection of C57BL/6 mice was performed by intraperitoneal injection of 100 trypomastigotes of the myotropic Colombian *T. cruzi* strain [20]. Groups of 10 chronic chagasic mice (6 months post-infection) were treated by intramyocardial injection with 10^6^ GFP^+^ CMSCs prepared in 50 μL of sterile 0.9% saline solution and injected into the lateral wall of the left ventricle by using an ultrafine needle (12.7 × 0.33 mm 29 G; BD Biosciences) guided by an echocardiography probe RMV 707B, 30 MHz (Vevo 707; VisualSonics, Toronto, ON, Canada). All animals were anesthetized by using an inhaled isoflurane anesthetic circuit 2% (Abbott) for 10 minutes before and after the intramyocardial injection. Control chagasic mice received the same volume of saline solution (n = 10). One mouse from the cell therapy group died during the intramyocardial injection. Therefore, the cell therapy group was composed of nine mice for the following analysis.

### Cell visualization by *in vivo* imaging system

Transplanted CMSCs were visualized by the *in vivo* imaging system (IVIS) Kodak Image Station 4000MM PRO equipped with a charge-coupled device camera (Eastman Kodak Company’s Health Group, now known as Carestream Health, Toronto, ON, Canada). For fluorescence imaging, the machine was configured for 550 nm excitation, 600 nm emission, 3 minutes of exposure, 262 binning, and fstop 2.5. The acquired images were analyzed with Carestream MI Application 5.0.2.30 software (Carestream Health). Whole body images were acquired from the ventral surface of the mice. Images were acquired immediately after intramyocardial transplantation, accompanied by an additional follow-up analysis 1 week later, in infected mice (6 months post-infection).

### Morphometric analysis

Heart sections were analyzed by light microscopy, and images were digitized by using a color digital video camera (CoolSnap, Montreal, QC, Canada) adapted to a BX41 microscope (Olympus). Morphometric analyses were performed 2 months after treatment by using software Image Pro Plus version 7.0 (Media Cybernetics). The number of inflammatory cells was determined by counting 10 fields (400×) per heart in hematoxylin-and-eosin-stained sections, and the percentage of fibrosis was determined in Masson’s trichrome-stained heart sections (200×) as previously described [3]. All of the analyses were blinded.

### Real-time reverse transcription polymerase chain reaction

Total RNA was isolated from heart samples with TRIzol reagent (Invitrogen, Molecular Probes, Eugene, OR, USA), and concentration was determined by photometric measurement. A High Capacity cDNA Reverse Transcription Kit (Applied Biosystems, Foster City, CA, USA) was used to synthesize cDNA from 1 μg of RNA in accordance with the recommendations of the manufacturer. Quantitative real-time reverse transcription polymerase chain reaction (qRT-PCR) assays were performed to detect the expression levels of tumor necrosis factor-alpha (TNF-α) (Mm 00443258 m1), interferon-gamma (IFN-γ) (Mm00801778m1), interleukin-6 (IL-6) (Mm00446190m1), cyclooxigenase 2 (COX-2) (Mm01307329m1), transforming growth factor-beta (TGF-β) (Mm00441724m1), and IL-10 (Mm00439616m1). The qRT-PCR amplification mixtures contained template cDNA, TaqMan Master Mix, and probes (all from Applied Biosystems). All reactions were run in triplicate on an ABI7500 Sequence Detection System (Applied Biosystems) under standard thermal cycling conditions. Non-template control (NTC) and non-reverse transcription controls (No-RT) were included.

### Confocal immunofluorescence analyses in heart sections of chagasic mice

The hearts obtained from chagasic mice were cryopreserved in Tissue-Tek (Sakura, Alphen aan den Rijn, The Netherlands), and 10-μm sections were obtained in Leica CM 1850 UV cryostat (Leica Microsystems, Wetzlar, Germany). To evaluate the presence of GFP^+^ cells in the heart, sections were incubated overnight with the following primary antibodies: anti-connexin 43 and troponin T (Santa Cruz Biotechnology, Inc.). On the following day, sections were incubated with Alexa fluor 594-conjugated anti-goat IgG or Alexa fluor 488-conjugated anti-rabbit IgG (Molecular Probes, Carlsbad, CA, USA). Nuclei were stained with 4,6-diamidino-2-phenylindole (DAPI) by using VectaShield Hard Set mounting medium with DAPI H-1500 (Vector Laboratories, Burlingame, CA, USA). Sections were then analyzed by using a FluoView 1000 confocal microscope (Olympus).

### Statistical analyses

All continuous variables are presented as mean ± standard error. Lymphoproliferation assay, morphometry, and qRT-PCR were analyzed by using one-way analysis of variance followed by Tukey’s multiple-comparison test with Prism 3.0 (GraphPad Software, San Diego, CA, USA). All differences were considered significant at *P* values of less than 0.05.

## Results

### Morphological and phenotypical characteristics of cardiac mesenchymal stem cells

**Figure 1:**
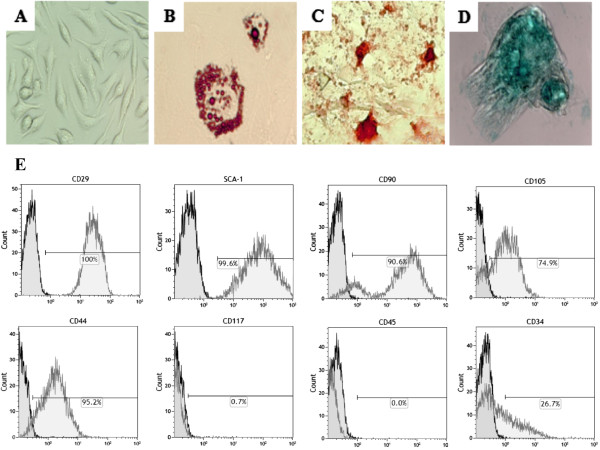
**Morphologic and multipotential characterization of cardiac mesenchymal stem cells (CMSCs). (A)** Phase contrast microscopy of CMSCs shows a fibroblast-like morphology in culture. **(B-D)** CMSCs were submitted to differentiation protocols into mesenchymal lineages. Cell differentiation was confirmed by positive staining with Oil red for adipocytes **(B)**, Alizarin red for osteocytes **(C)**, and Alcian blue for chondrocytes **(D)**. Magnification: 20×. **(E)** Flow cytometry analysis of CMSCs shows the percentages of CD29, CD90, CD45, Sca-1, CD44, CD105, CD117, and CD34 at passage 8.

Stem cells isolated from GFP transgenic mouse hearts had fibroblast-like morphologic characteristics of MSCs (Figure 1A). CMSCs were analyzed by flow cytometry to evaluate the expression of cell surface markers used to identify different cell populations (Figure 1E). A high percentage of cells expressing GFP was observed in the CMSC population. Additionally, a small percentage of CMSCs showed expression of CD117, whereas the majority of the cells expressed MSC markers (Sca-1, CD44, CD73, and CD90). In contrast, the percentage of cells expressing the hematopoietic cell marker CD45 was low in the passage used (passage 8), whereas the expression of CD34 remained high even at passage 8 (26%).

### Adipogenic, osteogenic, and chondrogenic differentiation potential of cardiac mesenchymal stem cells

CMSC cultures lacking differentiation stimuli maintained the undifferentiated state (Figure 1A). Stimulation of CMSCs with adipogenic differentiation medium for 15 days induced the accumulation of intracellular lipid, as shown by Oil red solution staining (Figure 1B). After 14 days of culture in osteogenic differentiation medium, cell clusters from CMSCs underwent osteoblast differentiation, as revealed by staining with Alizarin red 2%, showing the presence of calcium deposits in the cultures (Figure 1C). Twenty-one days after culture of CMSCs in chondrogenic differentiation medium, we observed the initial formation of cell clusters stained with Alcian blue, confirming the production of proteoglycans and chondrogenic differentiation (Figure 1D).

### Endothelial cell and cardiomyogenic differentiation of cardiac mesenchymal stem cells

**Figure 2:**
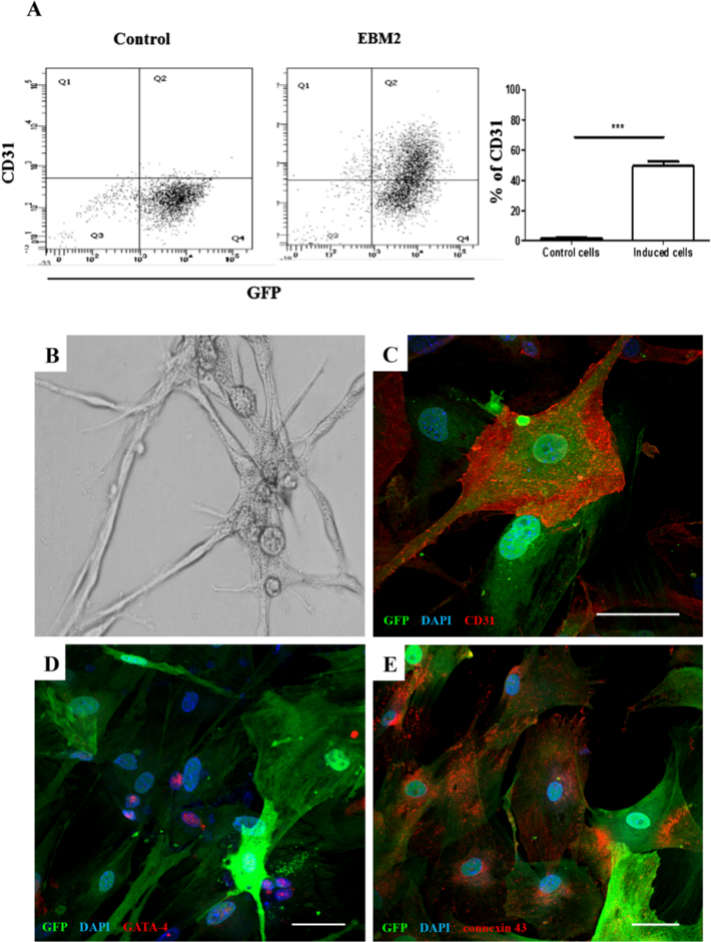
**Immunofluorescence, flow cytometry, and capillary-like tube formation analyses of cardiac mesenchymal stem cells (CMSCs) induced for cardiac and endothelial cell differentiation. (A)** Flow cytometry analysis of CD31 expression was evaluated in cells cultured or not with EGM-2 medium. An increase in the percentage of CD31 expression was observed in EGM-2 cell cultures. **(B)** Tubular-like structures of induced CMSCs were evaluated following culture on Matrigel for 24 hours induced by EGM-2 medium (magnification: 20×). **(C)** Confocal microscopy analysis of CMSCs after 10 days of endothelial induction by EGM-2 medium reveals positive staining for CD31 (red) in green fluorescent protein-positive (GFP^+^) CMSCs (green). **(D, E)** Four weeks after 5′ azacitidine induction, GFP^+^ CMSCs (green) began to express the cardiac transcription factor GATA-4 (red) **(D)** and the gap junction protein connexin 43 (red) **(E)**. Sections were co-stained with 4,6-diamidino-2-phenylindole (DAPI) (blue) for nuclei visualization. Bars represent 30 μm in **(B)** and 50 μm in **(D)** and **(E)**. *** p < 0.001.

We analyzed whether CMSCs cultured in EGM-2 developed an endothelial cell-like phenotype. Flow cytometry analysis showed an increase in the expression of CD31, an endothelial marker, after induction (Figure 2A). This was confirmed by confocal microscopy analysis (Figure 2C). CMSCs cultured for 10 days in EGM-2 were reseeded on Matrigel for another 24 hours. After this period, we observed the presence of capillary-like tube formation on Matrigel, confirming the endothelial cell differentiation potential (Figure 2B).CMSCs were cultured for 24 hours in the presence of the cardiomyogenic-inducing agent 5′ azacytidine. We did not observe any spontaneous beating of CMSCs stimulated or not with 5′ azacytidine. After 2 weeks of culture in the presence of 5′ azacytidine, we observed the expression of two cardiomyocyte differentiation markers, Gata-4 (Figure 2D) and connexin-43 (Figure 2E), by confocal microscopy analysis. However, we did not find an increase in troponin T gene expression by qRT-PCR analysis during the 3 weeks of induction with 5′ azacytidine (data not shown).

### Immunomodulatory potential of cardiac mesenchymal stem cells *in vitro*

**Figure 3:**
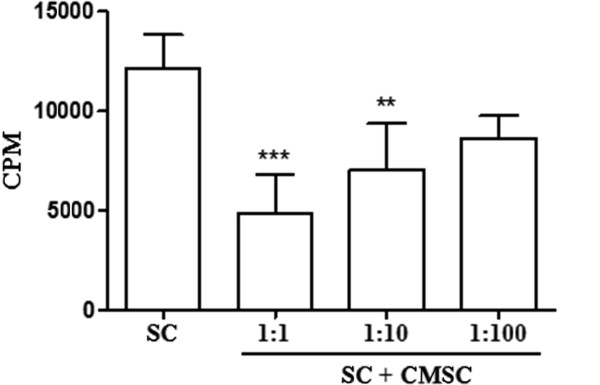
**Concentration-dependent inhibitory effect of cardiac mesenchymal stem cells (CMSCs) on concanavalin A-stimulated splenocytes.** Mouse splenocytes (SCs) were incubated for 2 days with concanavalin A in the presence of different numbers of mitomycin-treated CMSCs (CMSC/SC ratios of 1:1, 1:10, and 1:100). Spleen cell proliferation was assessed on day 3 by pulsing with ^3^H-thymidine for 18 hours. Values represent the mean ± standard error of the mean from four independent experiments performed in triplicate. CPM = counts per minute. ** p < 0.01, *** p < 0.001.

To analyze the immunomodulatory potential of CMSCs, we performed a lymphoproliferation assay by using splenocytes stimulated with the mitogen Con A in the presence or absence of CMSCs. As shown in Figure 3, the addition of CMSCs to the cultures of activated splenocytes caused a concentration-dependent inhibition of proliferation, demonstrating its immunosuppressive potential *in vitro*.

### Presence of green fluorescent protein-positive cardiac mesenchymal stem cells in the heart and expression of cardiac markers after intramyocardial injection in chronic chagasic mice

**Figure 4:**
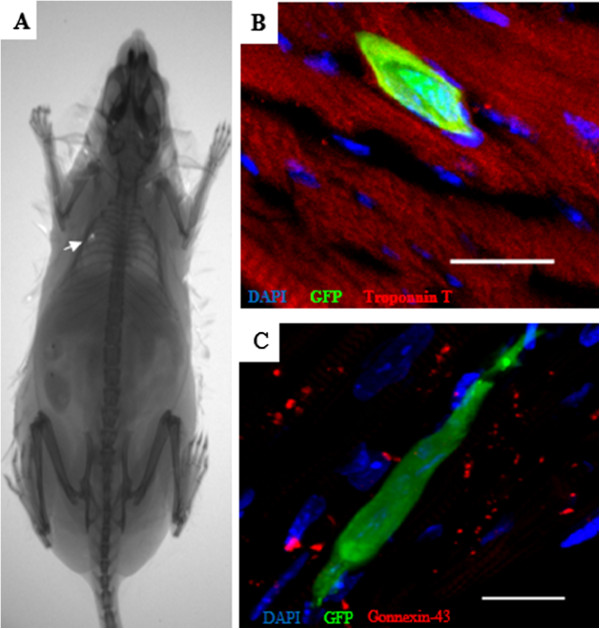
**Engraftment and survival of green fluorescent protein-positive (GFP**^**+**^**) cardiac mesenchymal stem cells (CMSCs) immediately and 1 week after cell transplantation in chagasic mice. (A)** The tracking of GFP^+^ CMSCs in chagasic mice was performed immediately in cell transplantation by using an *in vivo* image system. Arrow indicates a GFP^+^ fluorescent dot in the heart of a chagasic mouse after intramyocardial injection in the left ventricle wall. **(B, C)** Heart sections of CMSC-treated mice 1 week after intramyocardial injection show the engraftment and survival of GFP^+^ cells (green) in chagasic heart by confocal microscopy analysis. GFP^+^ cells in the tissue did not co-express the cardiomyocyte markers (red) troponin T **(B)** or connexin 43 **(C)**. Sections were co-stained with 4,6-diamidino-2-phenylindole (DAPI) (blue) for nuclei visualization. Bars represent 20 μm.

Transplanted GFP^+^ CMSCs were observed after intramyocardial injection guided by echocardiography. A fluorescent dot was visualized in the heart immediately after injection by using an *in vivo* imaging system in three of the nine analyzed mice, but no signal was detected in control mice (Figure 4A). One week after intramyocardial injection, *in vivo* imaging analysis was repeated and no signal was detected. However, GFP^+^ cells could be observed, 1 week after injections, in heart sections of CMSC-transplanted chagasic mice. However, none of the GFP^+^ cells observed co-expressed the cardiomyocyte proteins troponin T (Figure 4B) or connexin-43 (Figure 4C).

### Intramyocardial injection of cardiac mesenchymal stem cells modulates inflammation in chronic chagasic mice

**Figure 5:**
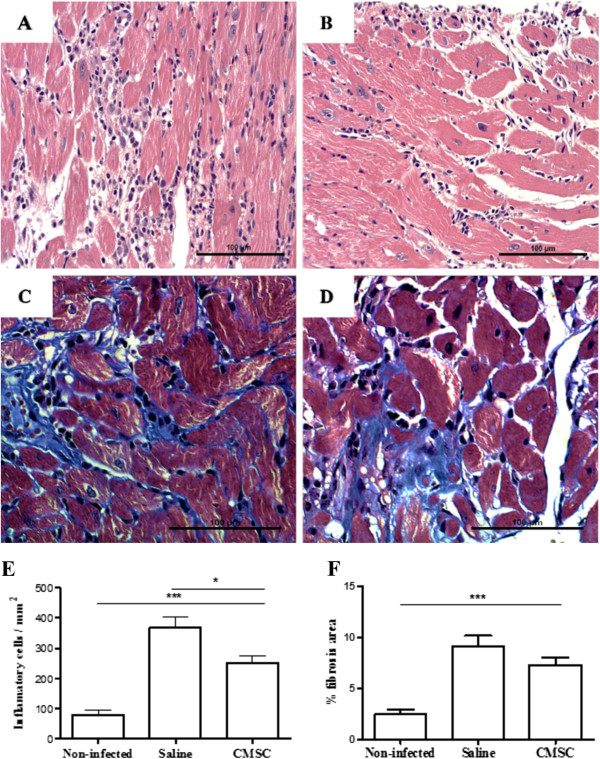
**Morphometric analysis of heart sections from uninfected and chagasic mice treated with cardiac mesenchymal stem cells (CMSCs) or saline.** Representative images of sections of hearts from mice euthanized 2 months after cell therapy with CMSCs and untreated controls are shown: heart sections of animals untreated **(A)** and treated **(B)** stained with hematoxylin and eosin (H&E) and heart sections stained with Masson’s trichrome obtained from untreated **(C)** and treated **(D)** mice. **(E)** Number of inflammatory cells per mm^2^ measured in sections stained with H&E. **(F)** Percentage of fibrosis quantified in sections stained with Masson’s trichrome. Results are expressed as mean ± standard error of the mean of 5 to 10 animals per group. *P <0.05. *** p < 0.001.

**Figure 6:**
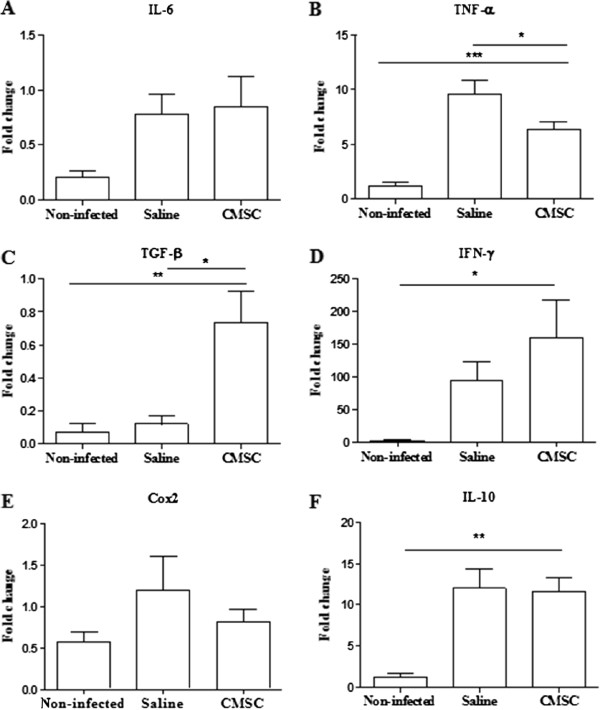
**Gene expression of inflammatory mediators in the heart.** Heart samples of uninfected or chagasic mice treated with saline or cardiac mesenchymal stem cells (CMSCs) were removed 2 months after therapy and analyzed by quantitative real-time reverse transcription polymerase chain reaction for the expression of interleukin-6 (IL-6) **(A)**, tumor necrosis factor-alpha (TNF-α) **(B)** (*P <0.05 versus saline and ***P <0.001 versus non-infected), transforming growth factor-beta (TGF-β) **(C)** (*P <0.05 versus saline and **P <0.01 versus non-infected), interferon-gamma (IFN-γ) **(D)** (*P <0.05 versus saline), cyclooxigenase 2 (COX-2) **(E)**, and IL-10 **(F)** (**P <0.01 versus non-infected).

Heart fibrosis and inflammation, typical features of chronic chagasic cardiomyopathy, were observed in *T. cruzi*-infected mice. Morphometric analysis demonstrated a significant reduction in the number of inflammatory cells, but not in the fibrotic area, 2 months after CMSC intramyocardial injection, when compared with saline-treated controls (Figure 5).Additionally, gene expression of pro-inflammatory and anti-inflammatory factors was evaluated in heart samples. Treatment with CMSCs produced a statistically significant reduction in the expression of TNF-α, but TGF-β gene expression was increased after CMSC transplantation (Figure 6B and C). No significant differences were observed in IL-6, COX-2, IFN-γ, and IL-10 gene expression between CMSC- and saline-treated groups (Figure 6A, D, E, and F).

## Discussion

Owing particularly to recent data suggesting their increased cardiomyogenic potential, heart-derived stem cells have been considered a promising source for use in cell therapy for cardiac diseases [14]. Additionally, MSCs have been isolated from the heart [7,14] by using previously protocols for cardiac fibroblast isolation [21].

In the present study, we isolated stem cells from the mouse heart and showed their mesenchymal features. The analysis of CMSC surface markers showed a pattern similar to that of MSCs obtained from different sources [22]. A small percentage of CMSCs presented CD117 (c-kit) expression. The low expression frequency of CD117 on cultured MSCs obtained from different organs has been previously described [23,24]. Similar characteristics have been described for fibroblasts obtained from different sources, and therefore unequivocal distinction between these two cell populations cannot be made [25].

CMSCs obtained in our study met additional criteria to be defined as MSCs, including differentiation potential into adipocytes, osteocytes, chondrocytes, and endothelial cells. These differentiation potentials are also observed in MSCs obtained from other sources, such as the bone marrow [22,26], and have been observed in additional CMSC studies [14]. However, when stimulated after previously described cardiomyogenic protocols, CMSCs expressed cardiac-lineage specifiers, such as GATA-4, and connexin 43, but failed to differentiate completely into beating cardiomyocytes *in vitro*. Moreover, after intramyocardial transplantation, we did not observe *in vivo* differentiation into cardiomyocytes at the evaluated time points. These findings are in contrast to previous reports describing the generation of differentiated cardiomyocytes *in vitro* by other populations of cardiac stem cells [14,27]. However, the efficiency of differentiation of MSCs into cardiomyocytes *in vitro* described previously is very low and has been constantly questioned [26,27]. Thus, the protocol of induction by using 5′ azacitidine has limitations in the induction of cardiomyogenic differentiation, which was confirmed in the present study.

Interestingly, we demonstrated for the first time that CMSCs have significant immunomodulatory potential both *in vitro* and *in vivo*. When co-cultured in the presence of splenocytes activated by Con A, CMSCs inhibited the lymphoproliferation in a concentration-dependent manner. Although this effect was not reported for cardiac-derived stem cells before, MSCs obtained from other sources, such as the bone marrow, have been well characterized regarding their immunomodulatory properties [28,29].

Furthermore, we demonstrated a reduction in the number inflammatory cells in the hearts of chronic chagasic mice after intramyocardial administration of CMSCs. Experimental infection with the myotropic Colombian strain of *T. cruzi* caused an intense inflammation composed mainly of mononuclear cells (macrophages and CD4^+^ and CD8^+^ T cells), which is one of the hallmarks of chronic chagasic cardiomyopathy [4]. Inflammatory cell modulation was accompanied by a reduction of TNF-α gene expression, but TGF-β, an anti-inflammatory cytokine, was upregulated. Previous studies have shown similar results with the use of MSCs obtained from adipose tissue and from bone marrow mononuclear cells [3,30].

Treatment with CMSCs, however, did not reduce the percentage of fibrosis. This may be explained by the fact that CMSCs contribute to scar formation by the abundant secretion of collagen type I deposited in the infarct area, as described by Carlson and colleagues [7] (2011). Moreover, in the present study, it is not possible to determine whether the beneficial effects exerted by CMSCs could be translated into improved heart function, since this experimental model is not associated with impaired heart function at the time points analyzed (unpublished data).

## Conclusions

CMSCs demonstrated an immunomodulatory potential in a Chagas disease model but did not reduce fibrosis or contribute to cardiomyocyte formation. These results suggest that MSCs may be beneficial in the context of Chagas disease cardiomyopathy. We speculate that, in combination with other cell types or factors, MSCs may participate in tissue regeneration.

## Abbreviations

CMSC: cardiac mesenchymal stem cell; Con A: concanavalin A; COX-2: cyclooxigenase 2; DAPI: 4,6-diamidino-2-phenylindole; DMEM: Dulbecco’s modified Eagle’s medium; FBS: fetal bovine serum; GFP: green fluorescent protein; IFN-γ: interferon gamma; IL: interleukin; MSC: mesenchymal stem cell; PBS: phosphate-buffered saline; qRT-PCR: quantitative real-time reverse transcription polymerase chain reaction; TGF-β: transforming growth factor-beta; TNF-α: tumor necrosis factors-alpha.

## Competing interests

The authors declare that they have no competing interests.

## Authors’ contributions

DNS contributed to conception and design, critical revision, data collection and analysis, and manuscript writing. BFS contributed to data collection and analysis, critical revision, and manuscript writing. CMA, JFV, and RHC contributed to data collection and analysis. MBPS and RRS contributed to conception and design and to critical revision. All authors read and approved the final manuscript.
